# Acknowledgment to the Reviewers of *Pathogens* in 2022

**DOI:** 10.3390/pathogens12020158

**Published:** 2023-01-18

**Authors:** Pathogens Editorial Office

**Affiliations:** MDPI AG, St. Alban-Anlage 66, 4052 Basel, Switzerland

High-quality academic publishing is built on rigorous peer review. *Pathogens* was able to uphold its high standards for published papers due to the outstanding efforts of our reviewers. Thanks to the efforts of our reviewers in 2022, the median time to first decision was 16 days and the median time to publication was 39 days. Regardless of whether the articles they examined were ultimately published, the editors would like to express their appreciation and thank the following reviewers for the time and dedication that they have shown *Pathogens*:
Aarti MishraKayvan EtebariAbdelfattah SelimKazuei IgarashiAbdol PaghehKazunori NagasakaAbdul GhafarKazuya MorikawaAbdul Razak MariatulqabtiahKei AmemiyaAbdulaziz AlhazmiKeisuke SuganumaAbel Villa-ManceraKeith ChappellAbelardo Silva-JúniorKelli BarrAbhay SatoskarKelly CondonAbhijit ShirkeKelly ElkinsAbhilasha Kandahalli Venkataranga NayakaKelly KnickelbeinAbhishek ChauhanKelsey E. LestebergAbhishek MishraKelvin G. K. GohAbhishek PrasadKen RosenthalAbid AliKenji GotohAbish StephenKentaro KatoAbraham Degarege-MengistKentaro MasujinAbraham PouliakisKeri N. NormanAd de GroofKerstin StinglAd VosKetki TendulkarAdam M. KayeKevin B. TemeyerAdam NockKevin BohannonAdam NovobilskýKevin E. LawrenceAdam SchrumKevin FonsecaAdebayo James MolehinKevin LooiAdil KhanKevin William ChristisonAdnan AkhterKeyvan PakshirAdnan MustafaKhadija AkaridAdrian BaranchukKhalid HameedAdriana AnitaKhalid Z. MasoodiAdriana BelasKhalil EslamlooAdriana CostaKhristine Joy C. AntiguaAdriana MieleKim O. GradelAdriana MorarKim Van VlietAdrianne N. EdwardsKirill AntonetsAdriano Cappellazzo CoelhoKirsty JensenAdriano CarrascoKirsty Le DoareAdrian-Valentin PotarnicheKíssila RabeloAgata KaczmarekKlaus-Peter HunfeldAgnès AubouyKobporn BoonnakÁgnes CsivincsikKoichi HashimotoAgnieszka BossowskaKoji WatanabeAgnieszka LejmanKomlavi Anani AfanouAgnieszka Perec-MatysiakKomwit SurachatAgnieszka Wiszniewska-ŁaszczychKonstantinos ArsenopoulosAgustín Olmedo-JuárezKosuke OkuyaAhlem JouiniKouji KimuraAhmad MirzaKovy Arteaga-LiviasAhmed BakryKrishna C. ChintaAhmed ElgazzarKrishna Kishore UmapathiAhmed ElmoslemanyKristen A. McLaurinAhmed HikalKristen PageAhmed KarmaouiKristin N. NelsonAhmed M. KheimarKristina E. M. PerssonAhmed MoustafaKrisztián FrankAhmed RakibKrithika RajagopalanAida DuarteKrunoslav BojanićÁislan de Carvalho VivariniKrupa NavalkarAistė BulavaitėKrystyna CwiklinskiAjay Kumar VijayKrzysztof GrzymajloAjay Suresh AkhadeKrzysztof TomczukAjitanuj RattanKrzysztof TrzcińskiAkash SabarwalKuang-Sheng YehAkebe Luther King AbiaKuldeep GuptaAkhalesh Kumar ShakyaKumiko YoshimatsuAkihiko MaedaKun LiÁkos MesterházyKun YangAla TaborKun-Hsien TsaiAlaa AtamnaKurt PfisterAlba Fresco-TaboadaKwanuk LeeAlba Marina GimenezKyu Chang ParkAlbert DescoteauxKyunam KimAlberto BorghettiLabrini V. AthanasiouAlberto DelbemLadislav ŠtěpánekAlberto EvangelistaLaia Bosch-CamósAlberto LázaroLakha SalaipethAldana VissaniLalit BatraAldo VenutiLampros FotisAlejandra Giselle Juárez-RebollarLarisa KarpenkoAlejandro A. SchäfferLars HusmannAlejandro Cabezas-CruzLaskarina-Maria KorouAlejandro DashtiLászló EgyedAlejandro Gómez-MejiaLászló FodorAlejandro Islas-JácomeLászló PalkovicsAlejandro Olivares-HernándezLaura Ambra NicoliniAlejandro Trijillo-GonzalesLaura DabosAlejandro VallejoLaura Dorina DinuAlejandro YañezLaura GoodmanAleksandra Kornacka-StackonisLaura Moreno-MesoneroAleksandra Maria KocotLaura Sánchez-MuñozAleksandra UzelacLaura TomassoneAleksandra WoźniakLaura WillenAlena ŽákovskáLauren A. SiltzAlessandra BarlaamLauren CharlesAlessandra De MatteisLaurent DeloozAlessandra Gutierrez De OliveiraLauretta TurinAlessandra HandisuryaLauro Velazquez-SalinasAlessandra NicolettiLavanya ChallagundlaAlessandra OcchialiniLavar KumarAlessandra PicardiLazzarini LorenaAlessandra RicelliLe Phuong NguyenAlessandro BartoloniLee NguyenAlessandro BonardiLeena AlhassanAlessandro F. T. AmaranteLeena MaunulaAlessandro GranitoLei HeAlessandro MalobertiLeigh KotzéAlessandro MinhoLeike ZhangAlessandro SinigagliaLénaïg HalosAlessia Di BlasioLenka SkalovaAlessio BocediLeny JoseAlexa M. LaheijLéo UchidaAlexander A. KirillovLeonardo BascoAlexander BolshoyLeonardo VelascoAlexander EckertLeonidas LotosAlexander I. AlexandrovLeopoldo PalmaAlexander KarasevLeslie OgorzalyAlexander KayLeszek GuzAlexander KrivoruchkoLetizia CeglieAlexander RobitzschLetizia Corinna MorlacchiAlexander V. GrishinLi YinAlexander V. ProninLia L. Lewis-XimenezAlexandra BeliavskaiaLiča LozicaAlexandra CorduneanuLidia Chitimia-DoblerAlexandra EhrensLidia PiechowiczAlexandra GambaryanLiesbeth FriasAlexandra J. MalbonLifeng ZhaiAlexandra ValenčákováLigen YuAlexandre LamasLigia Carla Faccin-GalhardiAlexandre LeitãoLihua WangAlexandro Rodríguez-RojasLijuan ZhouAlexandru BurlacuLi-Li ChenAlexey V. RakovLilia MessadiAlexios Vlamis-GardikasLilian SosaAlexis DziedziechLiliane MukaremeraAlexis Ribas SalvadorLin SongAlfonso NavasLina BinderAlfred O. AnkrahLinbo ZhaoAlfredo Diaz-LaraLinda BesterAlfredo Téllez-ValenciaLinda D. HazlettAli AbdallahLinda HouhamdiAli Al-AhmadLindsay TullochAli MazloumLing JinAli RamouzLinh Thuy NguyenAli Rezaei-MatehkolaeiLiqing RenAlica KocisovaLisa CrossmanAlice LytwynLisa HansonAlicia G. Marroquín-CardonaLisa KeithAlicia MasLisa LindesmithAlicja Wiercińska-DrapałoLisa WilliamsAlison LowLiselott Svensson StadlerAlla SplichalovaLisiane Morelia Weide AcostaAlphus D. WilsonLisle SnymanAlthea CampuzanoLister NicholasAltino Branco ChoupinaLivio TortaAlyssa GleichsnerLongxian ZhangAlyssa KoehlerLoredana FrascaAmanda C. TrimbleLoredana Sabina Cornelia ManolescuAmany MohamedLoredana SigilloAmber BarnesLorendane Millena De CarvalhoAmber JollyLorenzo PaceAmelia K. PintoLorenzo PolimenoAmelina AlbornozLorina Badger-EmekaAmer AlićLorraine Jones-BrandoAmin Talebi Bazmin AbadiLouis Pergaud SandjoAmina BasicLouise BasmaciyanAmir AbdoliLoukas KanetisAmit Kumar Singh GautamLourdes LledóAmit VikramLuan VuAmjad KhanLuana FioritiAmr GamilLuana Pereira Borba-SantosAmy GilbertLuba TchertanovAmy StrydomĽubica ĎudákováAmyn A. MalikLubomira Nikolaeva-GlombAna AfonsoLuca NalboneAna AguadoLuca VillaAna BocsanczyLucas Dos Santos DiasAna BudimirLucas MarquesAna Cláudia CoelhoLucia PallecchiAna Cristina FerreiraLucia PintilieAna CrnkovicLucia PotenzaAna DomingosLucia SignoriniAna HuertasLuciano Dos Santos BersotAna M. AlonsoLucie AmoureuxAna M. M. StoianLucie PaloqueAna Marcia de Sá GuimarãesLucie TamisierAna María Fernández-AlonsoLucienne TrittenAna María García-BoresLucinda J. BessaAna Maria PalomarLucio Diaz-FloresAna MorenoLucjan WitkowskiAna Patrícia Antunes LopesLucrezia BottalicoAna ReisLudwig SedlacekAna SantosLuigi Federico RinaldiAna VasićLuigi Segagni LusignaniAna Vujačić NikezićLuis Alberto Mejía-ManzanoAnamarija Jurčev SavičevićLuis Alberto Sanchez VargasAnanta Prasad ArukhaLuís CardosoAnanth Kumar KammalaLuís Fernando PariziAnanya Tupaki-SreepurnaLuis Martinez-GilAnastasia DiakouLuis Miguel GonzálezAnastasia SpiliopoulouLuis Orlindo TedeschiAnastasia VenierakiLuis ToroAnat FlorentinLuisa De MartinoAnca Oana DoceaLuisa MirandaAnders JohanssonLuisa NardiniAndré Alves DiasLuiz BermudezAndré Conceição PereiraLuiz Carlos KreutzAndré Lima AiresLuiz Daniel De BarrosAndré Luiz Rodrigues RoqueLuiz Felipe Domingues PasseroAndre Schutzer GodoyLuiz Shozo OzakiAndrea BattistoniLuiza Campos ReisAndrea BilgerLuka JurinovićAndrea De VitoLukas HegerAndrea FesslerŁukasz Augustyn PielokAndrea KressLuma AkilAndrea Micke MorenoLunda ShenAndrea MorrioneLutz Steffen GoehringAndrea SanchiniLuz E. De-BashanAndreas BurkovskiLynn DustinAndreas KrugerLynne T. BemisAndreas LeclerqueLynnette ArnoldAndrés Alonso Agudelo-SuárezLynnette C. GoatleyAndrés Felipe Opazo-CapurroLyric BartholomayAndres Jimenez Galisteo Jr.Ma Del Rocio Banos-LaraAndrés Mario VisintínMaciej FrantAndrew DiNardoMaciej GliniakAndrew HendersonMaciej KlockiewiczAndrew JinMaciej KochanowskiAndrew R. GorringeMaciej SkorackiAndrew R. MoorheadMadalena Vieira-PintoAndrew TeoMadhavi KakumanuAndrey GoginMagda RybickaAndrey KozlovMagdalena FlorekAndrey MukhinMagdalena KaraśAndrey ShadrinMagdalena RzewuskaAndrey SolovyevMahendran SekarAndrii SlonchakMahi M. MohiuddinAndrzej L. ChamieniaMahmoud E. KhalifaAneta NowakiewiczMai M. El-DalyAneta WoźniakMaira GoytiaAnette FriedrichsMaite OrruñoAngel H. AlvarezMaizbha Uddin AhmedÁngel Rodríguez-VillodresMakoto MatsubayashiAngela IvaskMalcolm Scott DuthieAngela M. Bosco-LauthMalek MarianAngela Melton-CelsaMałgorzata KępaAngela MontoneMalgorzata KrzyzowskaAngela Tellez-LanceMałgorzata Polz-DacewiczAngela WitteMalgorzata SulimaAni WidiastutiMalin SandbergAniello AnastasioMallikarjun BadadaniAnisuz ZamanMamoru NiikuraAnita M. KellyMana RaoAnita Q. GomesManas KotepuiAnita VillacísManas Kumar BagAnja JoachimManasi TamhankarAnja PecmanMangesh HadeAnkita PunethaManjusha LekshmiAnkita SrivastavaManon HoubenAnn NymanMansour A. AlghamdiAnn StockerMansureh GhavamAnna Arczewska-WłosekManuel De RojasAnna BajerManuel RitterAnna BeltrameMarat MakenovAnna CzarneckaMarc HerbAnna DobrutMarc JohnsonAnna Jakubska-BusseMarc KvansakulAnna LacastaMarc StadlerAnna LinkiewiczMarcello Otake SatoAnna M. SchotthoeferMarcelo GómezAnna MrzljakMarcia Cristina De Sena OliveiraAnna OlivieriMarcin BańburaAnna OrtuñoMarcin RóżalskiAnna PiekarskaMarco Di LucaAnna SkalniakMarco PalmaAnna V. Espinel-IngroffMarco PezziAnna WyrostekMarcos Cesar GonçalvesAnnamaria PassantinoMarcos Rogério AndréAnnarita BottaMarcus Tullius ScottiAnne KimballMarek AsmanAnne LavergneMarek WalczakAnne RidleyMargaret HosieAnne RousselMargarida DuarteAnne Sophie Le GuernMargherita Montalbano Di FilippoAnne SteneMarhiah C. MontoyaAnne-Claire LagréeMaria Alfonsa CavaleraAnne-Laure FavierMaria Angeles RojoAnnemarie E. LaumaeaMaria Bernabeu AznarAnne-Sophie PetitotMaria C. AlonsoAnne-Sophie Van LaereMaria C. BewleyAnnetta ZintlMaría CorreaAnnette MankertzMaría Cristina SosaAnnunziata GiangasperoMaria Da Conceição FontesAnoop AlexMaria De La Luz Galván-RamírezAnsari Mohammad AzamMaria DoligalskaAnshumali MittalMaria Elena MarcocciAnthi-Marina MarkantonatouMaría Eugenia GonzálezAnthony BowenMaria Eugenia Lopez-ArellanoAntoine M. DujonMaria ExindariAnton HartmannMaria Fernanda AchinellyAnton Pavlovych GerilovychMaría Florencia ViannaAnton SholukhMaria Francesca PiazzaAntonella CaniniMaria Grazia AmorosoAntonella FrassanitoMaría Guadalupe Frías-De-LeónAntonella MattanaMaria Isabel Nogueira CanoAntonella SchiavoneMaria João AlvesAntonieta Guerrero-PlataMaria João Castro GouveiaAntonino CataraMaria João PeixotoAntónio AbreuMaria João SantosAntonio Domenico MarsicoMaria Jorge Geraldes CamposAntonio FasanellaMaria José BorregoAntónio Gil CastroMaria José CaballeroAntonio Gregorio Dias JuniorMaria Josefa LombarderoAntonio Lozano-LeónMaria KantereAntonio Mario BulfamanteMária KazimírováAntonio MoriMaria Luísa LoboAntonio Paulo Gouveia AlmeidaMaría Martín-CuervoAntonio PlacidoMaria MascellinoAntonius (Tom) OomensMaria MazzitelliAnu NäreahoMaria Miklasińska-MajdanikAnuj AhujaMaría Moreno-del ÁlamoAnuli NjokuMaria OgrzewalskaAnurag ShuklaMaria PaparoupaAnusak KerdsinMaria PereiraAnwar KhanMaria PironAongart MahittikornMaria Rosario RodicioApurva Tadimari PrabhakarMaria ScaturroArancha Llama-PalaciosMaria Serena BeatoArévalo Sandra BarrosoMaria Teresa Galán-PuchadesAriadnna Cruz-CórdovaMaria Teresa ManfrediArianna CalistriMaría Teresa Martín-GómezArianna PuggioliMaria Victoria CardoArif RezaMaria-Dolores GarciaArif SiddiquiMaría-José ArgenteArifa KhanMarialetizia PalombaArifin Budiman NugrahaMariana Côrtes BoitéArkadiusz ZakrzewskiMariana SponchiadoArlind MaraMariano Sánchez CrespoArmanda RodriguesMariantonia MaglioArmando Andres Roca SuarezMariantonietta Di StefanoArnab NayakMariarosaria CampiseArnaud AvrilMarie DufresneArnold B. RabsonMarie-Anne BarnyArockiasamy Arun MiltonMarie‑Frédérique Le PotierArpan AcharyaMarie-Laure DardéArtem MetlinMariette F. DucatezArthur MorrisMarijana BasicArun PrasathMarina DiioiaArun S. KharatMarina LeiteArun Saravanakumar AnnamalaiMarina NisnevitchArunee AhantarigMarina NosikAsaf AchironMarina SofiaAshang LaivaMarinda MortlockAshenafi F. BeyiMarine WasniewskiAshish SrivastavaMario CrucianiAshok AspatwarMario Dante Lucio GiacobiniAshuin Kammar-GarcíaMario D’IncauAssunta BertacciniMario FargnoliAsta JudžentienėMario MardirossianAtef OriebyMario MasielloAthanasia XirogianniMario SantoroAthanasios GiatropoulosMariola BochniarzAtheesha SinghMariolina BrunoAthena AndreouMarion JostAthina S. TzoraMarion MigueresAurelie RakotondrafaraMarion WassermannAurora DiotalleviMarita GilljamAvinash PremrajMarius ColinAwad ShehataMarius MasiulisAyesha HassimMárius Vicent FuentesAyman El-BadryMariusz DylągAyush RanawadeMariusz MadejAyushi RaiMariusz NiemczykAzger DusthackeerMariyana AtanasovaAziz KarakayaMark F. WiserBabak PakbinMark J. GrygierBalamuralikrishnan BalasubramanianMark JohnsonBaldorj PagmadulamMark PeeplesBalik DzhambazovMark WunderlichBalint GergelyMarko MutanenBaoye HeMarkus KainulainenBarbara BacciMarlène ChevalierBarbara JonesMarta CanutiBarbara PaolettiMarta De LanaBarbara PieperMarta DecBarbara PintoMarta Denel-BobrowskaBarbara ZaapalaMarta KsiazczykBartłomiej FerraMarta PolinasBas Gerardus J. SurewaardMarta PotrykusBasile KamgangMarta VascellariBeata Koim-PuchowskaMarten John EdwardsBeata Labuz-RoszakMartha LegorretaBeata SadowskaMartin BartasBeata SzostakowskaMartín César-B. GutiérrezBeate KehrelMartin FayeBeatriz BrenerMartin GilbertBeatriz MaestroMartin JanickoBeatriz Vidana MateoMartin John DedicoatBecky HessMartín Pablo PorriniBen FanMartin SchmidtBen J. MaraisMartin TaylorBenedita Sampaio-MaiaMartina CrnogajBenjamin CullMartina KöhslerBenjamin M. LiuMartina LaidemittBenti GelalchaMartina MiluchováBernard KanoiMartina Srajer GajdosikBernard NaafsMartine Pestel-CaronBernardo Lopez-TorresMartine WallonBernd NeumannMárton JolánkaiBernd RodemannMaruti UppalapatiBernhard EllingerMarwa AttiaBernice HarrisMaryam DadarBertrand LeboucheMaryam Keshtkar JahromiBeth A. GarvyMarzena Rola-ŁuszczakBeth FuchsMasahiro AkiyamaBethany L. McGregorMasahiro OjimaBettie VoordouwMasanori MatsuiBettina SchulthessMasaomi KurokawaBettina WollankeMasfique MehediBhargav PatelMasmudur M. RahmanBiagio SantellaMasood Alam KhanBianca VezzaniMassimiliano GaldieroBijay KhajanchiMassimo CordaroBilali KabulaMassimo CristofaroBin WangMassimo PizzatoBingqian QuMateja OžanićBingqing XieMateus De Souza Ribeiro MioniBinu PrakashMateusz JankowskiBirgit SchrammMateusz M. PlucinskiBirgitte AndersenMathias MartinsBirke TewsMathieu GendrotBiruk Tesfaye BirhanuMatías CastellsBiswajit DasMatías Gastón PérezBjørn T. LunestadMatteo RiccòBo WangMatteo SenesiBogdan SoceaMatthew D. MooreBogusz Aksak-WąsMatthew DaughertyBojin BojinovMatthew J. MorganBonardi SilviaMatthew KnoxBoris A. FenioukMatthew L. FaronBoris A. YakobsonMatthew SpanglerBoris BienvenuMatthias DaubBoris VinatzerMaurizio MazzeiBoulouis Henri-JeanMauro Manuel Martinez PachecoBoyan GrigorovMauro PistelloBo-Young HongMax Carlos Ramírez-SotoBożena Futoma-KołochMaxim KhasnatinovBranislav VejnovicMaxime PichonBranka BedenićMay Lei MeiBrendan McMorranMaya RubtsovaBreno de Mello SilvaMayanka AwasthiBridget BarberMd. Abdus SoburBrigid Victoria TroanMd. AdnanBrock ArivettMd. Bashir UddinBruce MoltzanMd. Maidul IslamBruce S. SealMd. Rabiul IslamBruno Cuevas-ZuviriaMd. Saiful IslamBruno Martorelli Di GenovaMd. Tanvir RahmanBruno PontesMedhat MahmoudBryony WilliamsMegalie DossetBumsuk HahmMegan J. PuckelwartzByoung-Kuk NaMegan M. SloughÇağrı ErginMegan PennoCahyo BudimanMegan VogtCalin GhermanMehdi ElharrakCalogero Di BellaMehdi NateghpourCamila HamondMehdi OualhaCandace R. FoxMeir ShamayCapai LisandruMélanie IkehCara PagerMelissa Belló-PérezCarina CarvalhoMelissa J. CaimanoCarla CaliaMelissa Louise SykesCarla OplaatMenachem BanaiCarla PereiraMeng PuCarla ViegasMengmeng ZhengCarla ZannellaMeng-Shiou LeeCarlo ContiniMeredith B. BrooksCarlo Vittorio CitterioMeredith MorrisCarlos A. RossettiMerid Negash GetahunCarlos AlvesMeryem LemraniCarlos D. GrasaMeta SternišaCarlos E. SuárezMetka Pislak OcepekCarlos Felix MarinaMeysam SarsharCarlos GasparMiao-Chiu HungCarlos Hernando GómezMiaomiao ShiCarlos J. OrihuelaMichael ArabatzisCarlos Martínez-CarrascoMichael E. JohnsonCarlos MestresMichael GingerCarlos SaumellMichael J. WittyCarmelo A. Juárez-CastellóMichael KunzeCarmelo BonsignoreMichael LauzardoCarmen IscaroMichael LeschnikCarmen Patino-AlonsoMichael M. C. LaiCarmen PavelescuMichael MetzgerCarmen Rojas-MartínezMichael MinnickCarmine CoppolaMichael P. ReichelCarmona Encarnación MedinaMichael R. KnittlerCarola Sauter-LouisMichael RossCarolina DaviesMichael S. ChausseeCarolina Ripolles-AvilaMichael VaheyCaroline AmbergMichael WatsonCaroline ChaucheMichał GondekCaroline FreyMichal LinialCaroline SchneebergerMichel DionCaroline SubraMichel PélandakisCarrie BattenMichel PepinCarrie Mae LongMichela BertolaCarsten BalczunMichela DeianaCasey DroschaMichele GastaldelliCassiano Felippe Gonçalves-de-AlbuquerqueMichiel WijnveldCastillo Margarita MartínMiguel Ángel Ramos LópezCastro Carlos SandovalMiguel Ángel Sánchez-AlemánCatalina AvendañoMiguel FernándezCatherine A. GordonMihaela KavranCatherine BrissetteMihajlo ErdeljanCatherine E. VrentasMike RoseCatherine EichwaldMilad ZandiCatherine GuynetMilan KolarCatherine JaureguiMilana MitrovićCécile-Marie Aliouat-DenisMilica SelakovićCecilia AmbrosiMiloš StepanovićCecilia XimenezMinerva Gomez-FloreCédric MauprivezMing Jie LeeCeferino LópezMing YangCélia DechavanneMingguang FengCerline YangMing-Li ChenCesar GavidiaMing-Lung YuChandra Mohan SinghMingming LiuChandru SubramaniMing-Tse KuoChang-Chi LinMinli YouChanghoon ParkMinu ChaudhuriChaoqun YaoMinze ZhangCharith Raj Adkar-PurushothamaMiranda PittCharl VerweyMirela ImreCharlene KahlerMirja PuolakkainenCharles El-HageMirjam B. ZeiselCharles NfonMirko TrillingCharles W. ArmitageMiroslav BenićCharley Christian StaatsMiroslav GlasaCharlotte UetrechtMirosław MichalskiCharoonluk JirapattharasateMiroslawa DabertChase W. NelsonMiryam ValenzuelaChatanun EamudomkarnMisako KonishiChaturaka RodrigoMitsuyo KawaguchiyaChavez Adela OlivaMo D. SalmanChelsea DavisMohamad Fawzi MahomoodallyCheng-Chieh YenMohamed Abdo RizkCheng-I LeeMohamed LounisChenglei LiMohamed MeghilCheng-Maw HoMohamed ShaalanChengpeng FanMohamed ShaheenCheng-Wen LinMohamed ZommitiChen-Hsiang LeeMohammad Ali Al-DeebChenhua LiuMohammad DelghandiChenjian GuMohammad Javad AmanCheong Huat TanMohammad SadekuzzamanCheryl Ingram-SmithMohammad Shah JahanChi Keung Alvin LaiMohammad T. HedayatiChia-Min LinMohammad Tahir SiddiquiChiara IariaMohammed Mebarek BiaChi-Chien KuoMohammed Nadeem BijleChichun HoMohammed RohaimChih-Lin LinMohd AdnanChih-Yu ChiMohd Saeed SaeedChinedu O. EgwuMohd Zamri SaadChing-Hao TengMonica BalaschChi-Ping ChengMonica EmbersChishih ChuMonica Florín-ChristensenChitrita DebRoyMonica NunesChoaa El-MochtarMonica RagusaChongwen WangMonica SchützChris BosioMonika FajferChris FisherMonika MichaleckaChris H. L. LimMonika RinderChris LabakiMonika Szymańska-CzerwińskaChristelle Camus-BouclainvilleMontserrat ReyesChristelle Vauloup-FellousMoritoshi IwagamiChristian BurriMorris MaduroChristian CastilloMoshe Bar-JosephChristian HidalgoMostafa ElfawalChristian MengeMostafa RatebChristian PfallerMoulay Abdelmonaim El HidanChristiane SchneeMoyasar AlhamoChristina BergeyMubasher HussainChristina Li-Ping ThioMuhammad FarooqChristina VarveriMuhammad MohsinChristine JansenMuhammad N. ZahidChristine KeipertMuhammad NawazChristine SeersMuhammad SoyfooChristine Y. Y. WaiMuhammad Waqar AlamChristoph HörwegMuhammad YasirChristoph WelschMuhammad Zubair IqbalChristophe L. De-ChampsMuhammed DumanChristopher A. ClevelandMuhammet KarakavukChristopher A. RiceMunawwar Ali KhanChristopher C. EvansMunegowda KoralurChristopher NileMünir AktaşChristopher R. LittleMurugesan ChandrasekaranChristopher W. CutlerMustafa AlsarrafChristos ZamioudisMykola OvcharenkoChrysa VoyiatzakiMyron ChristodoulidesChrysoula G. OrfanidouNadeem UllahChuanchin HuangNadia El DibChunbin ZouNadia SaklouChungChun WuNadica KovačevićChunhe GuoNagendran TharmalingamChun-Ren WangNahla Galal MetwallyChyi-Liang ChenNana Kwadwo BiritwumCiavoi GabrielaNancy De BriyneCintia Hiromi OkinoNancy EndersbyCinzia SantucciuNancy FreitagClara BaldinNancy Weiland-BräuerClara MarínNanda Kishore RouthuClara ValeroNaomi S. TausClare WarrellNaouel KlibiClarissa BorgesNarcis Ioan PopescuClaudia Elena SotomayorNarit ThaochanClaudia EleniNatalia CheshenkoClaudia Fernández-AlarcónNatalia G. C. VasileiouClaudia FortunaNatalia KreshchenkoClaudia García-MoralesNatalia LomakinaClaudia KohlNatalia Mazur-PanasiukClaudia MarquesNatalia OsnaClaudia Parada MoraisNatalia RamosClaudia Paz SaavedraNatalie ElkheirClaudio CaprariNatallia V. DubashynskayaClaudio De LiberatoNathan J. BottClaudio Gabriel Lima‐JuniorNathan RothClaudio GenchiNathan W. CumminsClaudio MirandaNathaniel DenkersClaudio ValverdeNdawula Junior CharlesClaus MadsenNeelja SinghalClemax Sant’AnnaNeetu SrivastavaCollins K. TanuiNeha A. ThakreCôme ThieulentNejat DüzgüneşCommandeur SusannaNelson Rodrigo Da Silva MartinsConor O’HalloranNemeth MarkConrad FreulingNenavath Gopal NaikConsolato M. SergiNguyen Quoc KhuongConstantin CerbuNhung H. A. NguyenConstantina TsokanaNiccolo RiccardiConsuelo AlmazánNicholas JohnsonCorinne CuocNick KouttabCornelia HeinzeNick WheelhouseCory RobinsonNicola CarterCostanza VicentiniNicola FioreCourtney A. CookNicola PozzatoCraig HedbergNicola PuglieseCreanga AdrianNicolai DenzinCrina CotocNicolás GalarceCristiane GuzzoNicolas LévêqueCristiano CocumelliNicolas RuggliCristina Cacheiro-LlagunoNicolas TremblayCristina MesquitaNicole M. GilbertCristina NastasăNidal JaradatCristina ParolinNiels Nørskov-LauritsenCrystal BurkeNiels QvistCsivincsik ÁgnesNighat PerveenCsongor KissNikhat ParveenCynthia LordNiklaus H. MuellerCynthia OnzereNikol SulloCyril FersingNikolaos StilianakisDafne BongiornoNikolina RusenovaDag NymanNilakshi BaruaDalia Anwar HamzaNilanjan LodhDamien VitourNiloufar MahmoudiDamon P. EisenNima FirouzehDan Aaron YangNina WolfrumDana VanlandinghamNiroshini GunasingheDaniel BogemaNitin AmdareDaniel CameronNoemí Echegaray SuárezDaniel Gutiérrez-ExpósitoNoha AttiaDaniel HallinNomatter ChinganduDaniel Hernandez-PatlanNora PierangeliDaniel MatulićNorbert KissDaniel MorseNorhan K. Abd El-AzizDaniel P. DuranNorihiro HaradaDaniel Palmero-LlamasNorikazu IsodaDaniel SalamangoNurit EliashDaniel SantosNuttada PanpradistDaniel SteinOhad MazorDaniel WolfeOh-Deog KwonDaniel Z. P. FriedmanOkan AkhanDaniela AntolováOksana SytarDaniela CostaOleksii SkorokhodDaniela PintoOlga HeidingsfeldDaniela RandoOlga P. GavrilovaDaniele ChieffiOlga PawełczykDaniele GarcovichOlga S. SokolovaDanielle AdneyOlga V. MorozovaDanièlle Gunn-MooreOliver Chun Hung KwokDanijela ČerneOliver KroneDanijela TasicOlivia DorneanuDanila V. ZimenkovOlivier DiazDanilo Oliveira CarvalhoOlivier GlaizotDanny OttoOlivier SparaganoDante R. CulquiOmkar Vijay ByadgiDarci Moraes Barros-BattestiOndřej LenzDaria DanilenkoOnur CeylanDarinka VučkovićOpal PitaksakulratDariusz BartosikOrkide O. KoyuncuDarren W. WongOrtega-Pacheco AntonioDarta KlavinaOsamu KaminumaDaša StupicaOsbourne QuayeDat HaOskars KalejsDave GilmoreOsman AktaşDavid A. RasmussenOsman ErkmenDavid AlmenarOttmar HerchenröderDavid Emanuel Reyes-GuerreroOwen B. SpillerDavid EmeryOwen LeiserDavid González-BarrioOxana BelovaDavid Grove-WhiteÖzlem OyardıDavid J. McgeePablo González-MorenoDavid J. SullivanPablo MierDavid Lawrence SacksPablo Olivera FirpoDavid M. Brett-MajorPadet SiriyasatienDavid NegusPairot PramualDavid O’CallaghanPak Hin Hinson CheungDavid Perez PascualPalaniyandi KaruppaiyaDavid R. McGivernPallavi ChandraDavid R. McIlwainPam Dachung LukaDavid RotsteinPanagiotis KaranisDavid RoyPaniz JasbiDavide CossuPankaj DhakaDavide FirinuPanpan ZhouDavide SasseraPanpim ThongsripongDavide SoglianiPantelis KathariosDawei ZhouPao Theen SeeDean KonjevićPaola CociancicDebajit DeyPaola CremonesiDeepak KumarPaola GalluzzoDeepani FernandoPaola MuggeoDeepika RaiPaolo CapozzaDehua LaiPaolo MarracciniDelgado Patrick MathewsPaolo PozziDelia MunteanPapadopoulou OlgaDelphine Le RouxParag MaruDenis BourgeoisParamanandham KrishnamoorthyDenis E. KainovParide DioliDenis PiérardParisa K. KargaranDenis SerenoParissa TaheriDenis V. SudarikovPasquale PiconeDenis Victorovich TikhonenkovPat NuttallDenise BonillaPatrice GaurivaudDennis MuhanguziPatricia Agudelo-RomeroDervla T. D. KennaPatricia Arancibia-AvilaDessy RachmawatiPatricia CuervoDevadas KrishnakumarPatricia DiogoDevapriya SinhaPatricia GravesDhananjay YadavPatricia KönigDhruv DesaiPatricia M. LeglerDiana Gerardi ScorpioPatricia PesaventoDiana KataPatricia PriceDiana MartinsPatricia SeverinoDiane P. BartonPatricio Orellana-PalmaDiego AllonsoPatricio RamosDiego E. AlvarezPatricio Retamal MerinoDiego Francisco FacconePatrick HaningtonDiego QuirogaPatrick KellyDieter SteinhagenPatrick NjageDietmar SteverdingPatrick Stephan SebastianDimitra NtafouPatrizia CavadiniDimitri PoddighePatrizia ComoliDimitrios GougoulisPatrizia DanesiDimitrios SklirosPau Bosch-NicolauDimitris C. ChatzopoulosPaul A. FuerstDimosthenis ChochlakisPaul AirsDinesh MittalPaul ArnaboldiDion Charles MundyPaul AzzinaroDipayan BosePaul BloomDirceu RamosPaul H. DavisDivya BeriPaul M. SevernsDixit BhalaniPaul NguewaDmitriy Nikolaevich AndreevPaul W. J. TaylorDmitriy ShcherbakovPaula SantibáñezDmitry GryadunovPaulina KrawiecDmitry KireevPaulo Cezar CeresiniDmitry KostyushevPaulo Dell’Armelina RochaDmitry VerbenkoPaulo RoeheDoblin SandaiPavla Bartošová-SojkováDolly KhonaPavle BanovićDomenico FrezzaPaweł KrzyżekDominik ŁagowskiPaweł LechDominika SalamonPayam BehzadiDonald B. ThomasPedro FerreiraDonatas ŠneiderisPedro González-PechDonato Antonio RaelePedro J. BerzosaDongdong CaoPedro Mendoza De GivesDongmei LiPedro PellicerDonna SellersPedro RecheDoron TeperPedro ReisDorota Dwużnik-SzarekPei-Fen YenDorota WojniczPeiguo YuanDouglas GladuePei-Ling YuDouglas J. LaCountPenny A. RuddDouglas KerlinPer Moestrup JensenDouglas LeamanPerez Fernando Ivan FloresDragana GabrićPeriyasamy SivalingamDragomira MajhenPetca Razvan-CosminDuc Cuong BuiPeter BerlitDylan LeiPeter JarcuskaEamonn GormleyPeter KernEddie G. DominguezPeter LazarEdgar Mark WilliamsPeter MwangiEdgar Rodríguez-NegretePeter SchubertEdiane ZaninPeter WilsonEdinson Dante Meregildo RodriguezPetr HorákEdna YamasakiPetr MalýEdoardo CarrettoPetr PetrášEduardo Alvarez DuartePetr PyszkoEduardo BagagliPetra BandeljEduardo CostaPetra ȘurlinEduardo José Lopes-TorresPetros IoannouEduardo Rodríguez-NoriegaPhilip El-DuahEduardo Valencia-CanteroPhilip SchmidtEdward StephensPhilipp ArnoldEdwin BölkePhilipp IlinykhEdyta B. HendigerPhilipp J. PoxleitnerEelco F. J. MeijerPhilippe DufresneEfrem Alessandro FogliaPhilippe V. AfonsoEfrén Murillo-ZamoraPhillip S. CoburnEhdibaldo Presa-ParraPier Luigi FioriEhsan AhmadpourPiera Anna MartinoEiichi MomotaniPiera MazzoneEiji ArakawaPiera SoccioEike Roman HrinciusPierluigi RevegliaEileen RoulisPierre CappyEirini FilidouPierre DornyEkaterina ChernyaevaPierre RoquesEkaterina M. NestorovichPierre-Hugues StefanutoEkta MenghaniPiet Van RijnEleanor BurnettPiet W. J. De GrootElena BencúrováPietro BadagliaccaElena DalziniPietro FerraraElena GonellaPilar SolvesElena González-FandosPiotr B. HeczkoElena Marbán-CastroPiyanan TaweethavonsawatElena RizaPiyumali PereraElena ShipitsynaPolly Hang-Mei LeungElena UrcelayPoonam SharmaEleni G. KatsogiannouPoushali DasEleni GeorgakopoulouPovilas KavaliauskasEleni TopalidouPrabu GnanasekaranEleonora CataldoPradeep V. KadambiEleonora MauriziPradeepa JayachandranEliana MonteiroPrajna TripathiElias AlisaacPrakash Chand NegiElias PapadopoulosPrakash SahEliette BonnefoyPrakasha KempaiahElikaee SamiraPratima GuptaEline Pecho-VrieselingPravata K. PradhanElisa ZanottoPraveen Kumar BhartiElisabete FigueiredoPrem Lal KashyapElisabete MachadoPrerna DabralElisabeth LabruyerePrescilla Emy NagaoElisabetta RazzuoliPriscila IkedaElisabetta SuffrediniPriscila Mayrelle Da Silva CastanhaEliza WasilewskaPriscilla Maciel QuatrinElizabeth De GaspariPritesh DesaiEllen KnuepferPrzemyslaw CwynarEllen NisbetPutu Prathiwi PrimadharsiniElliot AndrophyQianru YangElmira JalilianQibin GengEloísa SevillaQingli DongElpidio Maria GarzilloQingquan ChenElsayed MehanaQingsen ShangElsherbiny ElsherbinyQingyun LiuElva Bonifácio AndradeQiuhong WangElvira AbolloQiwen DongElżbieta Hać-SzymańczukQuang Duy TrinhElżbieta Łopieńska-BiernatQuézia Moura Da SilvaElzbieta SarnowskaQutaiba AbabnehElżbieta ŻbikowskaR. Alan WilsonEman FayadRachel M. PalinskiEman KhalifaRachele CaglianiEmanuel GoldmanRachele GarellaEmanuele PalombaRachelle MendozaEmil ArbanasiRachid SabbahiEmília DvorožňákováRadha GopalaswamyEmilia HadziyannisRadoslaw LenarczykEmiliano MedeiRadu BotgrosEmilie BruezRafael Antonio Nascimento RamosEmilie GiraudRafael Calero-BernalEmilien JeannotRafael Coutinho Mello MachadoEmily A. KimminauRafael DelgadoEmily HoedtRafael NunesEmily M. EichenbergerRafail TasakisEmmanuel FavaloroRafał J. Pia̧tekEmmanuel OkelloRafał OgórekEmmanuel TetaudRaffaela PeroEmmitt R. JollyRaffaele ZarrilliEnder DinçerRahul D. JawarkarEndre JakabRahul Kumar TiwariEnedina Jiménez-CardosoRahul SuryawanshiEngin BerberRaimondo GaglioEngy ElekhnawyRaja Asad Ali KhanEnrico La PergolaRaja KalluruEnrico PavoniRajdeep BanerjeeEnrique MorionesRajiv PathakEnrique Reyes-NoveloRajneesh SrivastavaEnrique Rico-GarcíaRaju KhanEric AgboliRakel ArrazuriaEric L. BrownRaksawan DeenonpoeEric M. LeisRam Prakash RamanEric MosselRam SharmaEric P. F. ChowRamalingam BethunaickanErick Martinez-HerreraRamendra Pati PandeyErika S. HueRamiro López-MedranoErin E. SchirtzingerRamon GonzalezErna G. KroonRamona PalomboErnest ChiveroRan ZhuoErzsébet SándorRandall J. DeJongEsmeralda JuárezRandy E. SaccoEsperanza Duarte-EscalanteRanjan RamasamyEssi KorhonenRaphael B. StrickerEsteban GonzalezRaquel Villar-HernándezEstela Noguera-OrtegaRasmus GustafssonEsther LarreaRasoul MirzaeiEsther Monika SpeerRatko SukaraEsti Handayani HardiRatree TakhampunyaEthan L. MorganRaúl Pérez CaballeroEugénia de AndradeRavi KulkarniEugenia Silva-HerzogRavi Kumar KomaravoluEugenio ScanzianiRebeca Alonso-MongeEustachio TarascoRebecca FlahertyÉva KereszturiRedmond SmythEva KnuplezReed F. JohnsonEva TydénReed HolyoakÉva VárallyayReema SinghEvangelos AxiotisRegina PiresEvangelos I. KritsotakisReginaldo G. BastosEvgenii GusevRégulo López-CallejasEvzen BouraReham M. El-TarabiliEwa DługoszReham WasfiEwa DzikaReinout A. BemEwa JanczewskaRemigiusz GałęckiEwa Joanna Hanus-FajerskaRenata DezengriniEwa Jończyk-MatysiakRenata D’IncàEwa PaździorRenata Heisler NevesEwa TomaszewskaRenata ManconiEwa Więsik-SzewczykRenata Urban-ChmielEwan EadieRenate LuxEwan MacLeodRenate RankaEwan PlantRene KadenEwen C. D. ToddRenee M. TsolisFabian DeutskensRenuka N. AttanayakeFabian GiolittiRenyi LiuFabio MacchioniRey A. CarabeoFábio MendonçaRia BenkőFábio Muniz De OliveiraRicardo Antônio PolanczykFabio ZickerRicardo G. MaggiFabrice CompainRicardo Teresa RibeiroFabrizia VeronesiRicardo Wagner PortelaFabrizio BertelloniRicardo Wesley AlbercaFabrizio MaggiRichard A. BowenFadil BidmosRichard BinghamFahad KhanRichard BishopFaham KhamesipourRichard BowenFang WeiRichard D. DixFangzhu ZhaoRichard LavenFanny MathiasRichard MalikFany ReffuveilleRichard T. MarconiFarhat AfrinRick Twee-Hee OngFarrukee RubaiyeaRidi Rashika ElFátima AmaroRikio KirisawaFatima CerqueiraRikky RaiFatima Conceição-SilvaRita M. TraxlerFatma EldemeryRob SiebersFatme MawasRobert BransfieldFederica Di CesareRobert D. RaffanielloFederica LoiRobert E. MolestinaFederica MagagnoliRobert HarrisonFederica ObberRobert HarrodFederica ValerianiRobert HeinzenFederica VerraRobert KasprzakFederico ArmandoRobert KupczyńskiFederico RosaRobert McLeanFeixue WeiRobert StryińskiFélix Javier SangariRobert TrigianoFélix ValcárcelRobert WojtyczkaFeng CaiRoberta BattistiniFengjui ChenRoberta Maria FachiniFernanda Nazaré MorgadoRoberta MarquesFernando BauermannRoberta PedrazzaniFernando CardosoRoberto ChiesaFernando Esquivel-GuadarramaRoberto PaganelliFernando FerreiraRoberto PuleioFernando LealRobiatul AdawiyahFernando Nogueira De SouzaRobin KöckFernando ReyesRobin MacDiarmidFeyisara Eyiwumi OniRobyn HallFilipe Pereira-DutraRocío ChecaFilippo FratiniRod RussellFinazzi GuidoRodolfo M. De Moraes PeixotoFiona HenriquezRodolfo ParedesFiona Jane RadcliffRodrigo Almeida-PaesFiorenzo LupiRodrigo BaptistaFisayo OlotuRodrigo Cuiabano Paes LemeFlor H. PujolRodrigo De Paula BaptistaFlora De ContoRodrigo GigliotiFlora N. BalievaRodrigo MorchónFlorian H. H. BrillRodrigo RodriguesFloriana BonuraRodrigo Rosario-CruzFlorina S. IliescuRodrigo Saar GomesFonseca Maria Esther De NoronhaRoger A. Ramírez-BarriosFranca RossiRoger ChongFrances GullandRoger HewsonFrancesca AloisiRok ČivljakFrancesca De FalcoRok FinkFrancesca Di GiallonardoRokshana ParvinFrancesca DondersRoland BenzFrancesca GrippiRomain DuvalFrancesca RappaRomain Veyron-ChurletFrancesco Di GennaroRoman LeontovyčFrancesco FelizianiRomana MoutelíkováFrancesco FerraraRomana VulturarFrancesco MiraRon ShapiroFrancesco PrattichizzoRonald EllisFrancesco TiniRonaldo Cesar Borges GryschekFrancina Lozada NurRong FangFrancis B. NtumngiaRong-Nan HuangFrancis MuchaambaRonit SionovFrancisco ArnalichRosa AlduinaFrancisco Callejas-HernándezRosa Estela Quiroz-CastañedaFrancisco Cortés-BenítezRosa Maria Guillen FretesFrancisco EpeldeRosa ZampinoFrancisco Espinoza-GomezRosalind GilbertFrancisco Javier Pilar-OriveRosalyn E. PlotzkerFrancisco LlorenteRosamaria PennisiFrancisco MesaRosângela BarbosaFrancisco Murilo ZerbiniRosanna MarinoFrancisco Ponce-GordoRosario NicolettiFrancisco SautuaRosario SabariegosFranco Rubén RossiRose RaizmanFrançois TrotteinRosemary CaronFranjo MartinkovićRossella CacciolaFrank W. AvilaRossella PanareseFranklin ToapantaRoxanne A. CharlesFrans N. J. KooymanRoy Neal PlattFranz SellnerRoyo Francesc Xavier SorribasFranz ZinglRuan VeldtmanFred EyaseRuben AvendañoFrédéric A. C. J. VangroenwegheRuben Hernandez-AlcocebaFrederic ChevalierRubén Martín-EscolanoFrédéric CoutantRudi CassiniFrédéric GoormaghtighRudolf LüttickenFrédérique PasqualiRudy Caparros MegidoFumio SekiRuediger HauckFun-In WangRui-De XueFurhan IqbalRuifeng HuFuxuan WangRumyana MarkovskaFuyi WangRupesh KumarGábor ErdösRupinder KaurGabriel ParraRutger BennetGabriela CertadRuth C. ScimecaGabriela Chávez-CalvilloRuth TachezyGabriela GoujgoulovaRuvini PathiranaGabriela Jorge Da SilvaRuy JaureguiGabriela Loredana PopaRyan A. ReinkeGabriela MotoieRyan OliveiraGabriele ChilosiRyan TroyerGabriele PaganiSaber EsmaeiliGabriele PecherSabine BrandtGabriella GaglioSabine LichteneggerGabriella OrlandoSabine ZangeGaelle GonzalezSabri HacıoğluGaetano Magnano Di San LioSaccoccia FulvioGaetano OlivaSadiya ParveenGagandeep Singh SagguSaeed SadeghiGalya Ivanova GanchevaSaffiatou DarboeGamze YetişmişSaha PiuGannon C. K. MakSaharuetai JeamsripongGary SimonSaid OulghaziGavin GiovannoniSaito NobuoGayatri DevrajSakshi AroraGehad E. ElshopakeySalama Al-HamidhiGeoffrey HartSalvatore FrascaGeorg ConradsSamadhan J. JadhaoGeorge BelovSamantha HauGeorge BertsiasSamantha L. GrimleyGeorge Cosmin NadǎşSamantha TaylorGeorge EfthimiouSamar TharwatGeorge FthenakisSami AkbulutGeorge PelekosSami SimsekGeorge S. YapSamira GranadosGeorge T. TzirosSamuel DontaGeorge W. LiechtiSandeep TamberGeorge YendewaSandra AntunesGeorgiana DeakSandra D. AdamsGeorgios D. MichailSandra HalonenGéraldine PiorkowskiSandra Marcia MuxelGerardo Santos-LópezSandra PintoGerda KuiperSandra SoutoGerhard DoblerSandrine DahyotGerhard WunderlichSandro KlafackGermano CastelliSandro MatasGhazi KayaliSangmi LeeGhislaine MayerSanhita RoyGiancarlo RipabelliSanqiang GongGianluigi FranciSanthi DevasundaramGianmarco FerraraSanthosh RajamaniGideon WasserbergSantiago Mirazo VillarGilberto José De MoraesSanto PrevitiGilles BourgoinSantos Huarrisson AzevedoGilles GargalaSantos Marcos Veiga DosGilles ThuretSantosh GeorgeGimena DellapéSara BoboneGiorgio GallinellaSara GraggGiovanna GrimaldiSara VillariGiovanna PiccoloSarad PaudelGiovanni BeccariSarah AherfiGiovanni BocciaSarah El-NakeepGiovanni BubiciSarah GeogheganGiovanni CiliaSarah HardingGiovanni De Jesus MilanezSarah HendrickxGiovanni DeloguSarah JacksonGiovanni F. WidmerSarah LumleyGiovanni LanteriSarah Santos GonçalvesGiovanni SattaSaravanan SubramaniamGisela Canedo-MarroquínSari MattilaGiulia DegiacomiSarkar Mamta ChawlaGiulia PezzoniSascha TrappGiulia TarquiniSashi KantGiulia ZumboSasimanas UnajakGiuliano GarofoloSaskia WeberGiulio SeveriSatinder DahiyaGiuseppe BertoniSatoshi InoueGiuseppe LosurdoSatoshi MitaraiGiuseppe MancusoSatyavani KaliamurthiGiuseppe MarruchellaSaurabh MishraGiuseppe MigliaraSaverio PaltrinieriGiuseppe NascettiSaw BawmGiusto TrevisanSawsan A. ZaitoneGiusy CardetiSayaka MiuraGlauco Akelinghton Freire VitielloScavello FrancescoGlayciane Costa GoisScott J. WellsGleiston DiasScott LandfearGomes Gabriela SantosScott ParkerGómez Pedro A. JiménezSebastian GnatGomis Jorge RiveraSébastien GuyaderGonzalo German Cabrera VallejosSébastien HantzGonzalo Lopez-LorenzoSébastien ImbertGordana Blagojević ZagoracSébastien LambertGovindsamy VediyappanSébastien LhommeGraham BelshamSebastjan RadišekGrazia BossiSeiya SaitoGraziana Da RoldSelene ZárateGreg MatlashewskiSelma Soares De OliveiraGregor GrassSelwyn Arlington HeadleyGregorio CordónSenol PiskinGregory J. FonsecaSeong Beom ChoGregory M. AnsteadSeongbin ParkGregory SimonsSephra RampersadGreta VolpedoSepideh HosseiniporghamGrigoris AmoutziasSe-Ran JunGrissell TiradoŞerban Vifor Gabriel BerteşteanuGrzegorz KarbowiakSerena CavalleroGrzegorz LemańczykSergei SpiridonovGrzegorz PiechaSergey IordanskiyGuadalupe Guerra-SanchezSergey PustylnikovGuanghui WuSergey SokolovGudrun OvereschSergio E. BermúdezGuenther BuchholzSergio Gómez-RosalesGuerrino MacoriSergio O. AngelGuido RocchigianiSergio RosatiGuillaume BilodeauSérgio Santos De AzevedoGuillaume CharriereSergio Villanueva-SazGuillaume FichesSerra ÖrstenGuillaume GiraudSeung-Hun LeeGuillaume VelutSeungman ChaGuillermo Salgado MaldonadoSeverino Jefferson Ribeiro Da SilvaGulzhanat AimagambetovaSevero Vázquez PrietoGunjan AroraSeweta SrivastavaGuobing ChenSeyed Mohammad Kazem AghamirGustavo DelhonSezayi ÖzübekGustavo FontechaShadma W. AbdulwahabGustavo MontiShahana MajumderGustavo Rodrigues Makert Dos SantosShahzad AliGyan Prakash MishraShakeel MowlaboccusGyanendra GongalShamsaldeen Ibrahim SaeedGyörgy SiposShanlin KeHaim LevyShanmuga MahalingamHaiyan SunShaoting LiHala HusseinSharmila FagooneeHaley AdcoxSharon ClouthierHall Jennifer VanoverSharon Tirosh-LevyHamid BokharyShashwati MathurkarHamza AvciogluShauna KaplanHan MeiSheila DonnellyHana BandouchovaSheila V. GrahamHangjun KeSherif ElkafrawyHanish JainSherif HassanHanki LeeShigefumi OkamotoHanna JöstShigeharu SatoHanna MoniuszkoShigeki KamitaniHans-Peter FuehrerShigeru MatsumuraHanwei JiaoShigetoshi EdaHany M. R. Abdel-LatifShih-Yen LoHao ChengShih-Yi PengHao LiShilpi JainHapuarachchige Chanditha HapuarachchiShimi MeleppattuHarant HannaShingo NakamotoHarini SooryanarainShin-Ichi ItoHarold J. G. MeijerShipra SharmaHarold SalantShiv BharadwajHarun AlbayrakShivananda KandagallaHassan ChoudryShixing YangHassan HakimiShokoofeh ShamsiHåvard BjørgenShruti YadavHayato MasuyaShuichi SuzukiHeba Ahmed AbdallaShukho KimHeba Jafar SabbaghShüné V. OliverHediye Nese CinarShunping DingHedwin Kitdorlang DkharShuntai ZhouHee Il LeeShuyi MaHeidi GoethertShyunji HayashiHelen FogartySian FrosiniHelen IrvingSiciliano GiuliaHelena Gil AzinheiraSigrid C. RobertsHelena NejezchlebováSijun ZhengHélène MarchandinSilke R. KleeHelle Bielefeldt-OhmannSilmara Marques AllegrettiHelmut SattmannSilvana BeloHenrietta VenterSilvana ScarcellaHenriette Van HeerdenSilvia BianchiHenryk KrukowskiSilvia BonettaHerbert AuerSilvia FacciniHeriberto FernandezSilvia M. UriarteHerman K. EdskesSilvia PreziusoHernan Diego FolcoSilvia S. ChiangHernández-Velázquez Víctor ManuelSilvia Stefania LongoniHester DoyleSilvina Elena GutiérrezHicham El AlaouiSilvina FernandezHicham El-CostaSilvina L. AriasHideo WadaSilvio HemmiHideto FukushiSilvio PaoneHideyuki KanematsuSimon CleggHin Hark GanSimona GeorgievaHira L. NakhasiSimona SagonaHiroaki OkamotoSimone GalloHiroko YamashitaSimone MorelliHiroshi TomodaSimone PelettoHirotomo KatoSina BavariHo-Joon ShinSiok-Fong ChinHolger WilleSiraj Ahmed KhanHolly HughesSirpa RäsänenHonglei SunSit TimHongliang ChaiSita WithersHongsheng HuangSk Mohiuddin ChoudhuryHongzhuan WuSławomir ZduńczykHortense PetatSmaoui SlimHsiao-Hsuan KuoSmritee DabeeHuaiqiu HuangSneha RatnapriyaHuan DongSneha SinghHuanmin ZhangSnežana TomanovićHudson Alves PintoSoares José António Baptista MachadoHugh Robert WatsonSobhi F. LamlomHugo B. Barrios-GarcíaSofia AnastácioHugo FragaSofia CostaHugo SoudeynsSofya K. GarushyantsHuria MarnisSoham GuptaHyuk-Joon KwonSohyun ChoI. G. TiganovaSoibam Khogen SinghIain LamontSolange Bresson HadniIan DrySomya AggarwalIan FishSonia AlmeriaIan ScottSonia Lozano-SepulvedaIan SutherlandSonja J. OlsenIara LinharesSonsiray Alvarez-NarvaezIbne K. M. AliSoojin JangIbolya AndrásSophie Von DobschuetzIbrahim AbbasSoren UldumIbrahim M. SayedSotiris AmillisIgor BuzalewiczSpencer DurhamIgor DumicSreekumar OthumpangatIgor LoncaricSridhara KunjetiIkbal Agah InceSrijon BanerjeeIker A. SevillaSrikanth ManamIkram GuizaniSrinivas NellimarlaIlaria PatiSrinivas Reddy PallerlaIleana Adela VacaroiuStamatis KarakonstantisIlya GordeevStefan MagezIlya GordeychukStefania GallettiIlya TitovStefania VaraniImaan BenmerzougaStefano PetriniImmaculata XessStefano PileriInaki TiradosStefano RealeIndranil SamantaStefano SainasIndrė LipatovaStefano StracquadanioInes AranaStephanie LongetInês BártoloStephen A. KaniaInês L. S. DelgadoStephen A. KlotzInés ZulantayStephen CrookeInge LarsenStephen KlotzIngrid PapajováStephen LuInna SekirovStephen MelvilleIoana Adriana MateiStephen PageIoana Paştiu AnamariaStephen WoodwardIoanna PyrriSteve DunhamIoannis GiantsisSteven GoudyIoannis KosmasSteven P. DjordjevicIoannis PanagouliasStorm Blas MartinIoannis TheologidisStuart A. ThompsonIoannis-Dimosthenis AdamakisStuart DowallIppokratis MessaritakisSuat SarıbaşIra JainSubash SadIrena Vardić SmrzlićSubha DasIrena Wojsyk-BanaszakSubhadip ChoudhuriIrene Lo CignoSudhaker DharavathIrina BuchovecSudhir NavatheIrina TsitkoSudip DuttaIrina-Georgeta SufaruSudip K. GhoshIrineu De Brito JuniorSue NangIrshad SharafutdinovSufang LiuIrving E. SalitSumit MukherjeeIsabel Loreto Barroso De CarvalhoSuncana Simonic-KocijanIsabel MauricioSuncanica Ljubin‐SternakIsabella Dib Ferreira GremiaoSundharraman SubramanianIsabella GrishkanSunghyun YoonIsabella MarchesiSungil JangIsabelle CheminSunil D. SarojIsabelle DietrichSunil MoreIsabelle TurbicaSurajit BasakIsaia SymeonidouSuresh PantheeIshara S. ManawasingheSuresh ThanguduIsidro García-MeniñoSusan CorkIskren KaftandjievSusan M. NohIslay RodríguezSusan Madison-AntenucciIsmail NabilahSusan RobertsonIsrael NissanSusana AlaricoIstván EgerszegiSusana María Martín-OrúeItzel Amaro-EstradaSusana R. SousaItzel López-RosasSusana RemesarItziar Chapartegui-GonzálezSusanne SchulzIuliana NenuSusu ZughaierIván Herrera-PecoSutas SuttiprapaIvan PalaginSutherland MaciverIvan PavlovićSuvi TaponenIvan RaveraSuzy M. TeutschIvan S. KholodilovSvetoslav Nanev SlavovIvan V. BogdanovSwastik PhuleraIvana GobinSwati JainIvana KlunSwati JaiswalIvana KušanSwee Shan HueIvana MarekovićSwinburne AugustineIvana ŠimićSyazwan SaidinIvet YordanovaSylvia TwerskyIvo IvićSylwia Wdowiak-WróbelIvona MladineoSzu-Cheng KuoIvone MartinsTaeyong KwonIwona GabrielTaihei ItoIzabela Korona-GlowniakTaís Fukuta CruzIzabela Maurício De RezendeTaiwo AremuIzabela Podgórska-KryszczukTajana FilipecIzumi MashimaTakaaki KomaJ. Stephen LodmellTakashi AzumaJ. Venkatesh PratapTakashi KudoJacek BaniaTakashi KumagaiJacek FrancikowskiTakeshi KawaharaJacek NyczTalita ResendeJacek ŻmudzkiTamara P. RussoJackie MaharTamara SzentiványiJackson Victor de AraújoTamás MarikJacob LorenzoTanay BoseJacqueline V. SarrattTania CrucittiJacquelyn BowerTânia RosadoJacques CabaretTanja GerlzaJader De OliveiraTanja PessiJaeson CallaTanmoy ChakrabortyJae-Won ChoiTanushree DangiJagdish KhubchandaniTanya StratevaJaime Bustos-MartínezTao JingJajah FachirohTapan BhattacharyyaJake WhangTara Chand YadavJakub RuszkowskiTarmo NiineJakub SuchodolskiTaro IkegamiJames BoganTaru S. DuttJames C. KileTarun KeswaniJames CollinsTatiana A. SemenenkoJames Craig ForrestTatiana BetákováJames DoubTatiana Castro Abreu PintoJames J. ValdésTatiana RochatJames LaCourseTatjana Souza Lima KeesenJames R. CarterTatsufumi UsuiJames W. WilsonTatsunori NakanoJames WilliamsTatsuo KandaJamille G. DombrowskiTatyana PasechnikJamshid RazmyarTaufan BramantoroJan BocianowskiTeng J. PengJan E. ConnTengguo LiJan KopeckýTerence OdochJan NechwatalTeresa CoutinhoJan VotypkaTeresa Letra MateusJan WeberTeresa LettiniJanaína Capelli-PeixotoTerre Jasmina CasalsJanaina De Freitas NascimentoTerry A. KleinJane FreemanTetsuo AsaiJane K. DeverThanapong ChampahomJane MegidThapana ChontananarthJanet DalyThavasimuthu CitarasuJanet WhaleyThavy LongJanina P. LewisThibault MesplèdeJanovec VaclavThibeaux RomanJaqueline Mendes De OliveiraThillaiampalam SivakumarJaroslav SalavaThirumal RajJaroslav VadlejchThokur Sreepathy MuraliJarosław WaloryThomas ChambersJasim BasheerThomas CribbJason BodilyThomas J. OrtonJason FarlowThomas KlimkaitJason KimataThomas MelendyJavier ChinenThomas RomigJawhar GharbiThomas SvobodaJaya DantamThomas WattsJaya RajaiyaTiago NazarethJayedul HassanTiantian JiangJayne C. HopeTiberius BalaesJean MclainTiffany M. WolfJean Pierre KabueTim BullJean-Bernard DucheminTim LysykJean-François MagnavalTim PagetJean-Louis DacheuxTim SecottJefferson VaughanTimm HarderJeffrey BaraTimothy KeifferJeffrey GilbertTimothy R. HooverJeffrey J. AdamoviczTimothy SatterleeJelena PrpicTin PhanJelena PrpićTinatin DoolotkeldievaJenifer TurcoTing-Yu ChengJenna E. AchenbachTiny Motlatso HlokweJennifer BarrTiong Kai TanJennifer K. KetzisTiziana FioreJennifer KlutschTiziana ZottiJennifer L. ReimcheTobia PrettoJennifer N. WalkerTobias RupprechtJenny Chaparro-GutiérrezTobias TenenbaumJenny ShieldTodd OsmundsonJens André HammerlTohru SuzukiJens KrethTolstenkov OlegJens StrohaekerTomas KacergiusJeremy V. CampTomáš MacháčekJerko HrabarTomasz KulikJerome GouttenoireTomasz M. KarpińskiJerome Le NoursTomasz SzewczykJerry W. SimeckaTomislav MeštrovićJerzy GrabińskiTommaso FelicettiJesica Allyn HerrickTomohide MatsuoJesper WaldenströmTomohiro OishiJessica CanterTomoko KobayashiJessica L. JonesTorfinn MoldalJessica Maria AbbateTorgny SunnerhagenJesús Alonso Panti-MayToshifumi OhkusaJesús HernándezToshio HattoriJèsus Maria Perez JimenezTrim LajqiJesus Omar Muñoz-BelloTrudy G. MorrisonJesús RequenaTsung-Ying YangJesús Rodríguez-DíazTsung-Yu HuangJevtić RadivojeTsutomu OmatsuJhih-Hang JiangTsvetelina BatsalovaJia XueTsvetelina VelikovaJiachen HuangTuan L. PhanJiafen HuTuija GaddJiahao PengTzu-Chuan HoJianfeng LinUgo CovaniJianxuan WuUlf MagnussonJiaxin LingUlises De La Cruz-MossoJiayi WangUlrich H. FreyJiayu LiaoUlrike KemmerlingJiayue YanUlrike S. DiesterbeckJieshi YuUlvi GursoyJigang YinUrsula PanznerJih-Jin TsaiUrszula NawrotJimmy D. DikeakosUwe BraunJing HanVaihere DelauneJingen ZhuValentina Arsic ArsenijevicJinlong HanValentina BaccoliniJinxin ZhaoValentina CirkovicJiří ČernýValentina Laura DonatiJiri DreslerValentina MarcheseJiří SalátValentina MastrantonioJitender P. DubeyValentina MonisteroJitendra K. BiswalValentina PucaJitendra KumarValentina TagliapietraJoachim MorschhauserValentines ChisuJoachim RassowValeria MicheliJoachim SpergserValérie A. GeoffroyJoan Puig-BarberàValerija GromaJoan Tarradas FontValério Monteiro-NetoJoan Torras-AmbròsValesca AnschauJoana SimõesVan G. WilsonJoanna DąbrowskaVanessa Lievin-Le MoalJoanna DłużniewskaVangelis EconomouJoanna HildebrandVânia Luiza Deperon BonatoJoanna KuliszVanner BoereJoanna PulawskaVasco MenconiJoanna Skórko-GlonekVasia S. MavrogianniJoanna ZawitkowskaVasile CozmaJoanne M. PinkVasiliki E. FadouloglouJoAnne OliverVassilis PromponasJoao Aristeu Da RosaVassilis SkampardonisJoão Guilherme De Moraes PontesVázquez María Sol AriasJoão H. P. M. SantosVeerachai ThitapakornJoao Mello-VieiraVeerupaxagouda PatilJoão R. MesquitaVenkat ChaparaJoaquim Orlando Lima CerqueiraVenkatramana KrishnaJoaquín QuílezVera SadykovaJoe JamesVerde Sandra CaboJoe S. MymrykVerena SpieglerJoel RovnakVeronica Jimenez OrtizJohan H. MelendezVeronica SpinelliJohan SteylVeronika MaurerJohanna E. FraserVesna MilicevicJohannes SteffenVesna ŽupunskiJohannes WöstemeyerViatcheslav A. MordvinovJohn Asekhaen OhioleiVicky Van SantenJohn BarlowVictor AsensiJohn BennettVictor Dahl MathiasenJohn Bernet JohnsonVíctor M. RiveraJohn CoiaVictoria WatsonJohn D. PerryViet LeJohn DaltonVijay Kumar SharmaJohn HortonVikram ThakurJohn J. HalperinViliam ŠnábelJohn Lok Man LawVilla SimoneJohn McGivenVille HoikkalaJohn PappVinayak JoshiJohn W. NicholsonVincenzo Di Marco Lo PrestiJohnannes Jacob De SoetVincenzo ZammutoJolanda J. C. VoermansVinod Vikas PatilJolanta Behnke-BorowczykVinoth Kumar VenkatesanJolanta KutkowskaViola IntroiniJonas Nascimento CondeVioletta ZającJonathan FinkelVirginie BelletJonathan GriffithsVishwanath VenketaramanJonathan R. HoneggerVitalii TimofeevJonathan S. MarchantVittorio DibelloJonghoe ByunVittorio MazzarelloJordan MetcalfVittorio SarcheseJordi FiguerolaVivek SharmaJorge Alberto CortesVivian K. O’DonnellJorge BlancoViviane De Cássia OliveiraJorge García-HernándezVladimir KaberdinJosé Alberto Montoya-AlonsoVladimir SavicJose Alejandro Martinez-IbarraVladimir V. ZarubaevJosé AlmendralVolker GurtlerJosé AlundaVolker KrömkerJosé Angelo Lauletta LindosoVolker RickertsJosé António Baptista Machado SoaresWael ElfeilJosé Brites-NetoWael HegazyJosé Bruno MalaquiasWaldemar KazimierczakJose Francisco Ruiz FonsWalid AzabJosé Luis Chávez-ServiaWander Rogério PavanelliJosé Luis Díaz-HernándezWanderley De SouzaJose Luis RamirezWanderley Pereira OliveiraJosé M. BautistaWan-Long ChuangJosé Manuel Jaramillo OrtizWannaporn IttiprasertJose María RequenaWassim DaherJosé PérezWayne HeaselgraveJosé Ramos-VivasWei ChenJosé Roberto Machado-SilvaWei Guang SeetohJosé Sebastián DambolenaWei LiJose ThekkiniathWei SiJosep M. SierraWeisheng CaoJoseph FalkinhamWellington Pinheiro Dos SantosJoseph OrtegaWen Han TongJoseph PrescottWenjun ZhuJosh ColstonWenliang XiangJoshua GurtlerWen-Yu ChuangJoshua KamaniWeronika GonciarzJoshua Oluoch AmimoWieslaw SwietnickiJosipa HabušWilfried HaasJoško RačnikWilhelm GernerJovana SadlovaWill K. ReevesJovanny GomezWilliam A. BowerJoy SturtevantWilliam SchwanJózsef KónyaWilliam WintJózsef SókiWilliam ZaragozaJuan AyalaWinka Le Clec’hJuan C. Vázquez-UchaWioleta Chajȩcka-WierzchowskaJuan F. Espínola-NoveloWioletta Adamus-BiałekJuan Hernandez GarciaWisit KaewputJuan Pablo VicoWitold SurewiczJuan VesgaWojciech WakulińskiJudit SymmankWojciech ZygnerJuhee AhnWolfgang BäumerJulia AlmeidaWolfgang SchweigkoflerJulia CritchleyWolfgang SiposJulia GonzálezWolf-Rainer AbrahamJulia HilliardWon Gi YooJúlia JarošováWon Hyung ChoiJulia Monjarás FeriaWynand GoosenJulia Rebecca PortXavier VallèsJulia RidpathXenia KostouliasJulia RomanoXiangjie SunJulia SommerXiangli ZhaoJulián Ruiz-SáenzXiao ChenJuliana Quero ReimãoXiaohui ZhouJuliane LokauXiaolong LiJulie ArsenaultXiaomao WuJulie FoxXiaomeng LiuJulie GuignotXiaonan ZhangJulien AndréaniXimeng SunJulien Van GrevenyngheXin HuJulio C. GarciaXin YangJulio Plaza-DíazXinhai YeJulio Rodriguez-AndresXinlei LiJulio Vicente Figueroa-MillánXinran LiuJun HuangXin-Ru WangJun WangXisha WangJun ZhouXue Zhi ZhaoJung-Eun KimXupeng HongJunhao ZhuYafeng QiuJun’ichi YoshidaYanan LiuJunki MaruyamaYang QiuJunko AkadaYang ShenJunya YamagishiYanina Paola HeckerJunyi JiaoYanli LiJunzo NorimineYanmin WanJuraj NemecYannick RossezJustin A. PachebatYaoyu FengJustin RobyYash GuptaJustina PradaYasuyuki GotoJustyna StruzikYasuyuki NagasawaJyotsna ShahYi-Chang LiaoKabiru AkinyemiYi-Hsien LinKai Sen TanYi-Jung ChangKai Siang ChanYike ShenKai TaoYing Kai ChanKai XuYing Zhang (Yunnan University)Kaice LaFaversYing Zhang (Zhengzhou University)Kaisu Rantakokko-JalavaYlva PerssonKaitlin ForsbergYoann S. Le BretonKaixin LiangYohan NguyenKajal GuptaYohan N’GuyenKallol DasYong ZhouKálmán ImreYongcai LiKalyan K. DewanYongchang CaoKamal A. M. Abo-ElyousrYongfen XuKamenna VutovaYongjin QiuKamila Rybczyńska-TkaczykYoshihisa HashiguchiKandahalli Venkataranganayaka AbhilashaYoshikazu Honda-OkuboKannimuthu DhamotharanYoshimitsu FujiiKanok PreativatanyouYoshiro KobayashiKarel HruskaYosra A. HelmyKaren BorgesYounes LaidoudiKaren Du PreezYoung Sang AhnKaren JohnstoneYoussef A. AttiaKaren L. SternYoussef AachouiKaren LaurieYraima CordeiroKaren PohYuan-Ti LeeKaren PowerYubhisha DabasKaren V. Pineda-HidalgoYucen XieKarin StiasnyYu-Chung ChangKarl BechterYulema ValeroKarl SeydelYuling XieKarla BitencourthYung-Fu ChangKarolina KotYunus Emre BeyhanKarrie RoseYuriy L. Vasil’evKashi Raj BhattaraiYusen Eason LinKatalin Magyar-TáboriYusuf AbbaKatalin SalankiYusuf OmosunKatarína PastirčákováYvonne Adu-AgyeiwaahKatarina ŠimunovićZachary B. MackeyKatarzyna Buńkowska-GawlikZachary WeinerKatarzyna Filip-HutschZackary FitzsimondsKatarzyna GarbaczZafar HandooKatarzyna Grzelak-BłaszczykZaheer AhmedKatarzyna Otulak-KoziełZakaria A. BakaKatarzyna PobiegaZamantungwa KhumaloKatarzyna SikorskaZarrin BasharatKatarzyna TołkaczZdzisław LaskowskiKate F. JohnsonZekun MuKateel G. ShettyZelalem Eshetu BekaluKateřina NedbalcováZemfira N. KaramyshevaKaterina RoubalovaZhanibek YessimbekovKatharina L. LohmannZhengjun WuKatharine E. MagorZhenyu ZhangKatherine GillZhi-An HuKatherine PflaumZhihao SunKathlyn LavalZhimin YinKathrin ArndtsZhiyou DuKathryn M. JonesZiarno MałgorzataKathy Ann Burek-HuntingtonZijian ZhangKatia PinelloZizheng ZhengKatie AldredZobia NoreenKatie GassZofia BakulaKatri JalavaZoi AthanasakopoulouKaushal Kishor RajakZoltán DeimKausik DattaZongdi FengKavita SharmaZongliang GaoKay Annette RamsayZsuzsa KalmárKay Choong SeeZuzana Macek Jilkova


